# Endometrioma Complicated by Tubo-Ovarian Abscess in a Woman With Bacterial Vaginosis

**DOI:** 10.1155/IDOG/2006/84140

**Published:** 2006-11-30

**Authors:** Shahryar K. Kavoussi, Mark D. Pearlman, William M. Burke, Dan I. Lebovic

**Affiliations:** ^1^Division of Reproductive Endocrinology and Infertility, Department of Obstetrics and Gynecology, University of Michigan, 1500 E Medical Center Drive, Ann Arbor, MI 48109-0276, USA; ^2^Department of Obstetrics and Gynecology, University of Michigan, 1500 E Medical Center Drive, Ann Arbor, MI 48109-0276, USA; ^3^Division of Gynecologic Oncology, Department of Obstetrics and Gynecology, University of Michigan, 1500 E Medical Center Drive, Ann Arbor, MI 48109-0276, USA

## Abstract

*Background*. Tubo-ovarian abscess involvement of an endometrioma has been reported in cases of patients with polymicrobial sources such as *Neisseria gonorrhoeae, Chlamydia trachomatis*, and obligate anaerobic bacteria; however, bacterial vaginosis (BV) predisposing to abscess formation in an endometrioma has not been reported to date. *Case*. Superinfection of an endometrioma was surgically diagnosed in a patient with known advanced-stage endometriosis after she presented with acute pelvic inflammatory disease symptoms and was unresponsive to antibiotic therapy. Gram-negative rods were cultured from the endometrioma. On admission, cervical, blood, and urine cultures were negative; BV was diagnosed on normal saline wet prep and gram stain. *Conclusion*. This case raises the possibility of BV ascension to the upper genital tract predisposing to abscess formation in endometriomas. Therefore, aggressive treatment of BV in patients with known advanced-stage endometriosis may be considered to prevent superinfected endometriomas.

## INTRODUCTION

Tubo-ovarian abscess (TOA) is a sequela of pelvic inflammatory disease (PID) that is comprised of an infectious, inflammatory complex encompassing the fallopian tube and ovary. The proposed pathophysologic mechanism for TOA development includes ascending infection as well as hematogenous and lymphatic routes [1]. The infectious source is typically polymicrobial and several reports have identified *Escherichia coli*, *Neisseria gonorrhea*, and *Chlamydia trachomatis* and a variety of obligate anaerobic bacteria as commonly associated microorganisms [1, 2].

Cases of TOA involving endometriomas have been reported in the literature, and women with revised American Society for Reproductive Medicine (ASRM) stages III-IV endometriosis [3] have been found to have an increased occurrence of TOA [2]. *E coli* was more frequently cultured from aspirated abscesses in women with concomitant endometriosis than if no endometriosis was present. In addition, previous pelvic surgery was found to increase the risk of TOA [2]. We report a case of a woman with stage IV endometriosis with a TOA involving an endometrioma, in the absence of cultured organisms, who had evidence of bacterial vaginosis (BV) on normal saline wet prep and culture.

## CASE

A 41-year old nulligravida presented to the University of Michigan (UM) Reproductive Endocrinology and Infertility clinic for an evaluation of a six year history of primary infertility. Her prior fertility evaluation and treatment history was significant for four previous cycles of ovulation augmentation with clomiphene citrate in conjunction with timed intercourse and a normal hysterosalpingogram several years earlier. Her initial workup at UM revealed normal ovarian reserve testing, normal values for TSH and prolactin, and her husband's semen analysis was normal. Transvaginal ultrasonography showed persistent bilateral 3.5 cm ovarian cysts that had a ground-glass appearance consistent with endometriomas [4]. The patient underwent laparoscopic evaluation due to the large endometriomas at which time she was diagnosed with stage IV endometriosis. Due to dense adhesive disease, the procedure was converted to an exploratory laparotomy. Three right ovarian endometriomas were removed, and the cyst wall of each was excised with subsequent cauterization of the bases for hemostasis. The left ovarian endometrioma was approached; however, it was not removed due to severe adhesions and the patient's desire for fertility. The patient's postoperative course was unremarkable.

Five weeks after surgery, the patient underwent a hysterosalpingogram which showed a normal uterine cavity, right tubal patency, and left hydrosalpinx without spillage of dye. She subsequently underwent two cycles of gonadotropins in conjunction with intrauterine inseminations, but had suboptimal responses.

**Figure 1:**
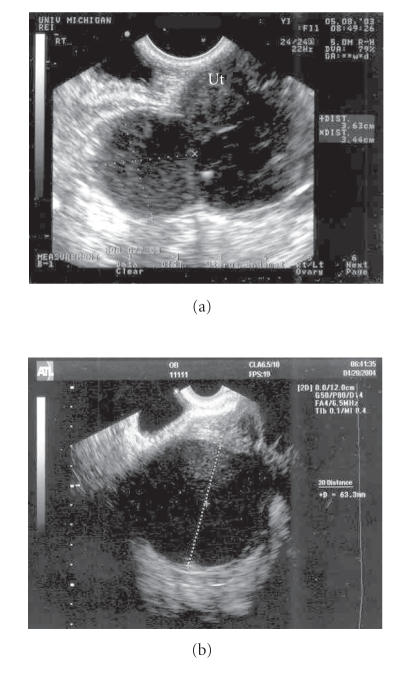
(a) Transvaginal ultrasound image, shortly after initial evaluation, of persistent bilateral ovarian masses adjacent to uterus (Ut). (b) Ultrasound image of left ovarian cystic structure after patient presented with symptoms of acute pelvic inflammatory disease.

Nine months after surgery, the patient presented to the emergency room with fever and abdominal pain. Her temperature was 38.7°C. Her abdominal and pelvic exams showed moderate tenderness, small amount of vaginal discharge and bacterial vaginosis as evidenced by clue cells on a normal saline wet prep. Gonorrhoeae and chlamydia cultures were obtained. Transvaginal ultrasound showed a 7 cm left adnexal mass with uniform echogenicity (Figure 1). Her WBC was 16.7 K. She was admitted to the hospital for intravenous antibiotic administration but despite broad-spectrum coverage, high-grade fevers continued. On hospital day 3, after discussion of risks, benefits, and alternatives that included conservative management, she was consented for definitive surgical management and underwent a modified radical hysterectomy with bilateral salpingo-oophorectomy and lysis of adhesions.

**Figure 2:**
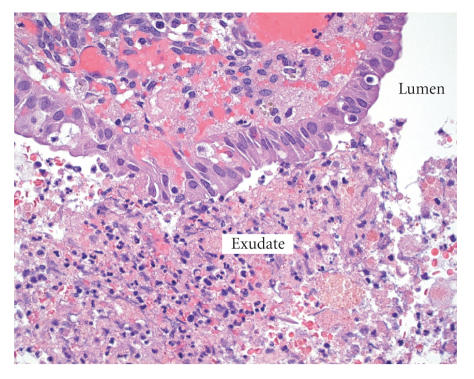
Endometrioma with associated edema, hemorrhage, acute inflammation, and marked eosinophilia (H & E, ×40).

Although all cervical, blood, and urine cultures were negative on hospital admission, the left ovary contained an ovarian abscess with gram negative rods within the left-sided endometrioma. Gonorrhoeae and chlamydia cultures were negative. There was an evidence of BV on normal saline wet prep and culture. Histology of the left fallopian tube and ovary revealed an endometriotic cyst with massive edema, hemorrhage, acute inflammation, and marked eosinophilia (Figure 2) with an associated pyosalpinx consistent with a TOA.

## COMMENT

The development of TOA among women with endometriomas may be due to an increased susceptibility to infection, [2] particularly in the altered immune environment seen with ectopic endometrial glands and stroma, [5] although there are no epidemiologic data available to support this theory. Previous surgical procedures involving the pelvic organs have been found to increase the risk of TOA formation in patients with endometriosis [2]. Interestingly, in this case report, the patient's surgery took place nine months prior to clinical presentation and the presence of acute inflammatory cells in the endometrioma makes the probability of a long incubation period or superinfection of a chronic infectious process unlikely but not out of the realm of possibility. There is one case report of a 7 month period of time from surgery to superinfected endometrioma [1]. Similarly, in terms of the timeframe of events, although an HSG performed in the presence of an apparently dilated fallopian tube carries an increased risk of pelvic inflammatory disease, [6] this patient's procedure was performed eight months prior to the onset of her acute symptomatology and histologic findings. There was no known change in partners since the patient's initial office visit until the time of presentation with symptoms of acute pelvic infection.

Although the association between BV and the increased risk of an ascending PID is well established, [7] in the present case, the finding of BV in the absence of isolated organisms on cervical, blood, and urine cultures raises the question as to whether BV that has ascended to the upper genital tract is a predisposition to abscess formation in an endometrioma. More study is necessary to investigate this matter and to elucidate whether aggressive treatment of BV in patients with known advanced stage endometriosis, akin to screening and treatment in patients who are to undergo hysterectomy to prevent vaginal cuff abscesses, should be considered to prevent super-infected endometriomas.
